# Exploring the role of KIR3DL2 on NK cells in hepatocellular carcinoma and its potential prognostic implications

**DOI:** 10.1016/j.isci.2024.110637

**Published:** 2024-08-08

**Authors:** Jie Zhu, Anli Jin, Baishen Pan, Wei Guo, Wenjing Yang, Beili Wang

**Affiliations:** 1 Department of Laboratory Medicine, Zhongshan Hospital, Fudan University, Shanghai 200032, China; 2 Department of Laboratory Medicine, Shanghai Geriatric Medical Center, Zhongshan Hospital, Fudan University, Shanghai 201104, China; 3 Department of Laboratory Medicine, Xiamen Branch, Zhongshan Hospital, Fudan University, Xiamen 361015, China; 4 Department of Laboratory Medicine, Wusong Branch, Zhongshan Hospital, Fudan University, Shanghai 200940, China

**Keywords:** Bioinformatics, Cancer, Immune response

## Abstract

Hepatocellular carcinoma (HCC) is a globally prevalent malignancy with a high recurrence rate, significantly impacting prognosis and survival. This study aims to identify prognostic molecular markers using single-cell sequencing of tumors and adjacent tissues in primary and recurrent HCC patients. We analyzed single-cell sequencing data from tumor and adjacent normal tissues of primary and recurrent HCC cases to compare immune cell quantity and gene expression profiles. Recurrent HCC patients exhibited a significant reduction in infiltrating NK cells expressing KIR3DL2. Pseudotemporal and cell communication analyses revealed these KIR3DL2^high^ NK cells were in a quiescent state, suggesting NK cell exhaustion and poor prognosis. KIR3DL2 expression in peripheral blood NK cells correlated with that in tissues, highlighting its potential as a prognostic marker for HCC.

## Highlights

• KIR3DL2 is significantly downregulated on NK cells infiltrating tumor tissues in HCC patients
• Reduced KIR3DL2 expression correlates significantly with poor prognosis in HCC patients
• KIR3DL2 on NK cells in peripheral blood shows its potential as a prognostic biomarker

## Graphical abstract

**Figure:**
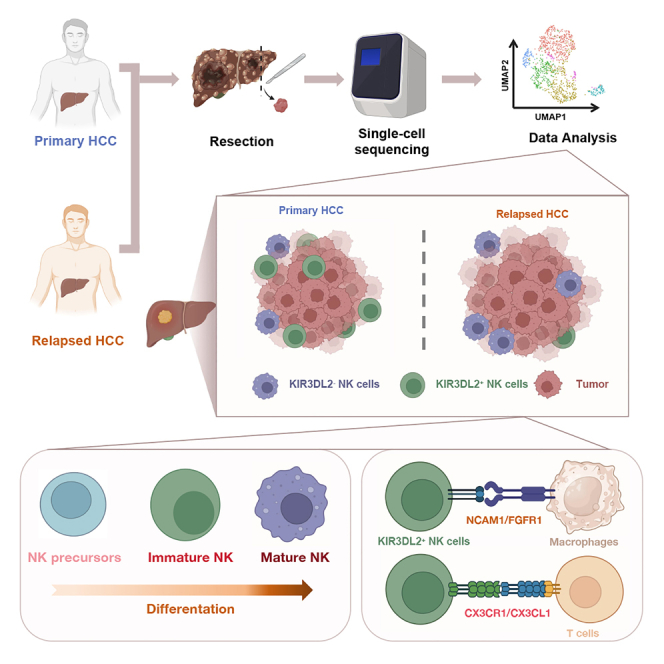

## Introduction

Hepatocellular carcinoma (HCC) is a highly prevalent tumor worldwide, ranking fifth among all cancers in incidence and fourth in cancer-related mortality. It represents an extremely malignant form of cancer.^1^ In recent years, significant progress has been made in the treatment of HCC, particularly with the clinical utilization of targeted therapies such as sorafenib and lenvatinib, leading to notable improvements in patient prognosis. However, despite these advancements, the recurrence rates among HCC patients remain persistently high. Studies indicate that following surgical resection for early-stage HCC, approximately 13% of patients experience recurrence. Moreover, those treated with local ablation face an alarming 70% recurrence rate within five years.^2^^,^^3^ The occurrence of HCC recurrence is a multifaceted process influenced by various factors, with changes within the tumor microenvironment playing a pivotal role. After treatment, alterations in the patient’s tumor microenvironment may occur, including shifts in immune cell populations, modifications in the tumor-associated matrix, and fluctuations in vascular density. These changes occasionally create an environment more conducive to the growth and spread of tumor cells, thus influencing the recurrence of HCC.^4^^,^^5^^,^^6^

Natural killer cells (NK cells) within tumor tissues may have a significant impact on HCC recurrence.^7^^,^^8^ NK cell is a type of immune cell capable of recognizing and killing tumor cells, representing a crucial component of the immune system. Studies indicate the potential infiltration of NK cells within the tumor tissues of HCC patients. Ideally, these NK cells can recognize and eradicate tumor cells, thereby restraining tumor growth and spread. However, sometimes, the recurrence of HCC might be linked to decreased activity and numbers of NK cells within the tumor microenvironment.^9^^,^^10^^,^^11^

KIR3DL2, also known as CD158k, belongs to the killer cell immunoglobulin-like receptor (KIR) family,^12^ considered as an inhibitory receptor predominantly identified on NK cells, and it has the capability to recognize shared epitopes present on HLA-B27, HLA-A3, and HLA-A11 class I glycoproteins within the major histocompatibility complex (MHC).^13^ Additionally, KIR3DL2 exhibits the ability to bind exogenous DNA containing CpG motifs, thereby modulating NK cell functionality in a context-dependent manner.^14^ Prior investigations have associated KIR3DL2 with various diseases, including ankylosing spondylitis and cutaneous T cell lymphomas like Sézary syndrome, CD30^+^ cutaneous lymphoma, and transformed mycosis fungoides.^15^^,^^16^^,^^17^ However, its specific involvement in HCC remains enigmatic. In this study, single-cell sequencing data from HCC tissues were scrutinized, comparing patients with recurrent HCC against those without recurrence. Notably, a conspicuous downregulation of KIR3DL2 expression was observed on NK cells within HCC patients experiencing recurrence. Leveraging insights from The Cancer Genome Atlas (TCGA) database, our endeavor aims to illuminate the nuanced molecular implications and clinical relevance of KIR3DL2 in the context of HCC recurrence.

## Result

### Processing of single-cell data and classification/naming of cell populations

**Figure 1:**
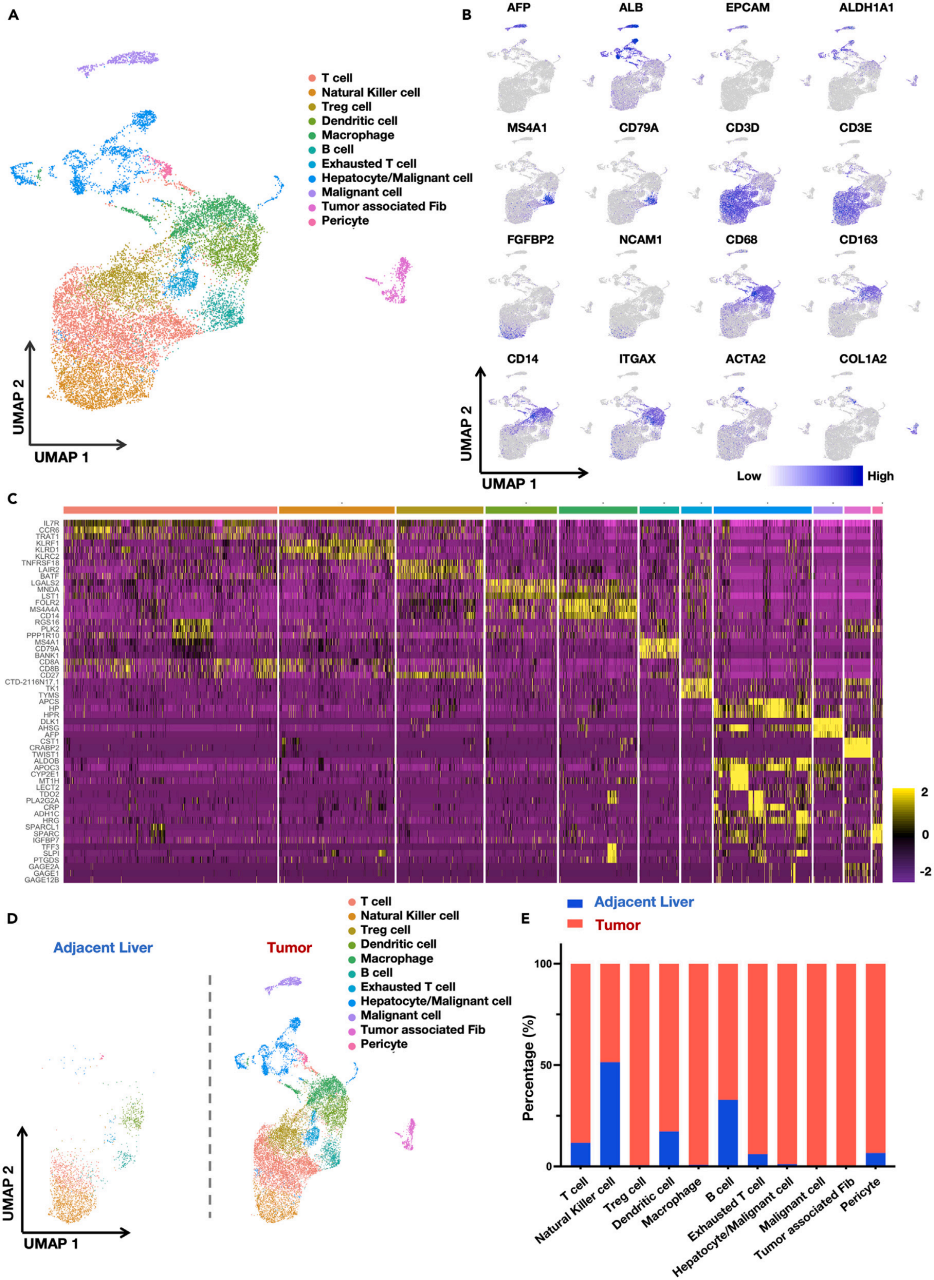
The profile of tumor tissue and adjacent non-tumor tissue microenvironment single-cell sequencing data in hepatocellular carcinoma patients (A) Single-cell sequencing data are presented in the form of UMAP plots to show the distribution of various cell types. (B) The cell-specific genes expression in UMAP plot. (C) Heatmap of the top three genes highly expressed in different cell clusters identified by single-cell sequencing. (D) Differential distribution of cells in tumor and adjacent tissues in UMAP plot. (E) The proportion of cells in tumor and adjacent tissues in histogram plot.

The single-cell data analyzed in this study were sourced from the China National GeneBank DataBase with the sample identification number CNP0000650. This dataset comprises single-cell sequencing information from a total of 16,498 cells extracted from liver tissues. These cells were obtained from tumor tissues and adjacent non-tumor tissues of 12 treatment-naive primary HCC patients and 6 cases of early recurrent HCC. To precisely classify cell types, we performed principal component analysis (PCA) on the data followed by dimensionality reduction clustering using uniform manifold approximation and projection (UMAP) (Figure 1A). Subsequently, CellMarker 2.0 (http://117.50.127.228/CellMarker/) was employed to input the top 20 highly expressed genes from each cluster generated after dimensionality reduction clustering into “Cell annotation.” This computational method facilitated the determination of cell names and types for each clustered group. Additionally, we analyzed the expression patterns of cell-specific genes (such as EPCAM, ALDH1A1, and ALB for epithelial and tumor cells; CD79A and MS4A1 for B cells; CD3D and CD3E for T cells; FGFBP2 for NK cells; ITGAM, CD33, CD68, CD14, and CD163 for monocytes and macrophages; ITGAX for dendritic cells; ACTA2 and COL1A2 for fibroblasts; and PECAM1 for endothelial cells) (Figures 1B and 1C). By integrating these analyses with the aforementioned computational outcomes, we made conclusive determinations regarding the cell types for each clustered group. Following the identification of cell types within each cluster, we conducted a comparative analysis of cell proportions and quantities between normal liver tissue and tumor tissue. The findings unveiled a significantly higher abundance of most cell types in tumor tissues compared to their counterparts in normal liver tissues, while the quantity of NK cells remains relatively consistent between tumor tissue and adjacent liver tissue. However, individual patient-specific analysis comparing tumor tissues with adjacent liver tissues indicated a substantial reduction in both the count and proportion of NK cells within the tumor tissue among the majority of HCC patients (Figures 1D, S1A, and S1B).

### KIR3DL2 exhibits significant downregulation in NK cells of early recurrent HCC patients

**Figure 2:**
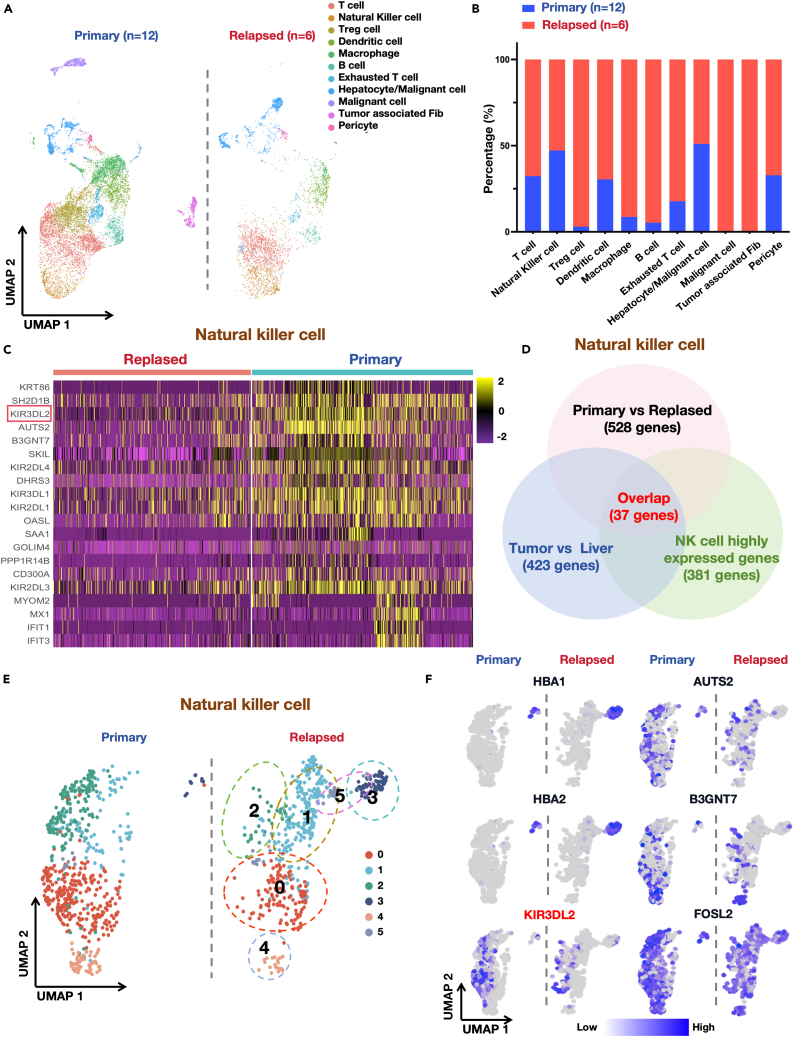
Single-cell sequencing data of the tumor microenvironment in patients with primary and relapsed HCC (A) Differential distribution of cells in tumor from patients with primary and relapsed HCC. (B) The proportion of cells in tumor from patients with primary and relapsed HCC in histogram plot. (C) Heatmap of gene expression differences in NK cells originated from tumor tissue between patients with primary and relapsed HCC. (D) The intersection of the three sets of differentially expressed genes, with a total of 37 genes in common. (E) The UMAP plot showing the clusters of NK cells from patients with primary and relapsed HCC. (F) The top six genes expression in UMAP plot.

We investigated the infiltration of NK cells within the tumor tissues of both primary HCC patients and those experiencing early recurrence. Strikingly, we observed no significant differences in either the proportion or quantity of NK cells between these two groups (Figures 2A, 2B, S1C, and S1D). Subsequently, we delved into an in-depth analysis of differential gene expression in NK cells from both cohorts. Figure 2C depicts the top 20 differently expressed genes in NK cells across the two groups. Notably, we detected substantial differences in the expression of KIRs among NK cells from the two cohorts, particularly KIR3DL2, KIR2DL4, KIR3DL1, KIR2DL1, and KIR2DL3. Of significance, KIR3DL2 exhibited the most pronounced expression difference between the two groups.

In an effort to elucidate the distinct expression profiles of NK cells between these cohorts, we intersected genes differentially expressed in NK cells from tumor versus non-tumor tissues, genes distinguishing early HCC recurrence patients from primary patients, and genes showing heightened expression within the NK cell population. This rigorous analysis revealed 37 genes, with KIR3DL2 ranking third among the differential genes (Figure 2D). Figures 2E and 2F showcase the UMAP dimensionality reduction plots depicting NK cell distribution and the expression patterns of the top 6 differential genes within NK cells, including HBA1, HBA2, KIR3DL2, AUTS2, B3GNT7, and FOSL2. Notably, KIR3DL2 demonstrated predominant expression in clusters 0 and 2, and compared to primary HCC patients. Notably, there is a significant deficiency of KIR3DL2-highly-expressing NK cells in cluster 0 among HCC patients with tumor recurrence. However, the distribution of KIR3DL2 within these two clusters is not entirely uniform, with partial expression of KIR3DL2 observed in cluster 0.

Overall, there was a significant decrease in KIR3DL2 expression within the NK cells of early recurrence patients.

### KIR3DL2 expression patterns in NK cell clusters within liver tumors

**Figure 3:**
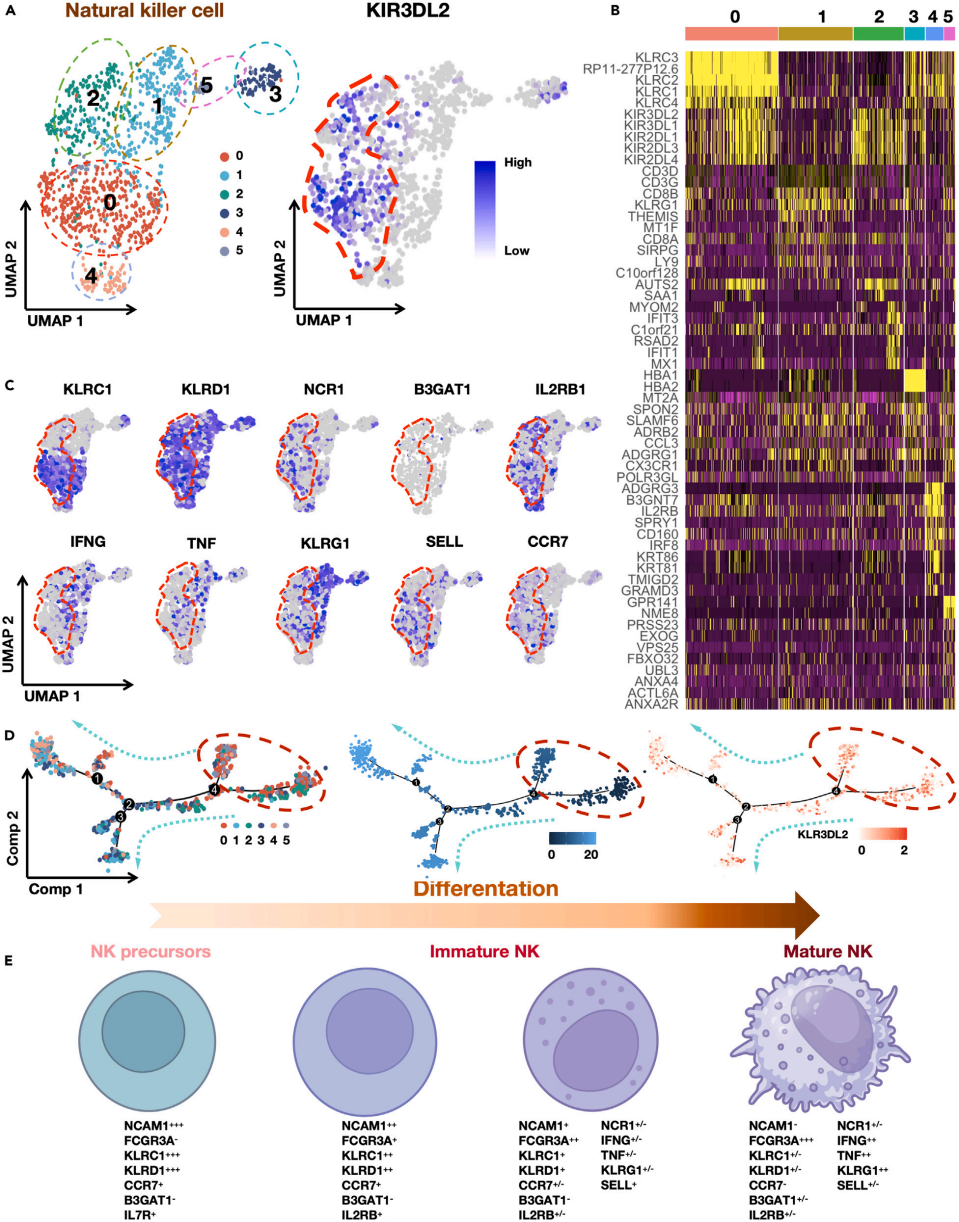
KIR3DL2 expression patterns in NK cell clusters within liver tumors (A) KIR3DL2 mainly expressed in cluster 0 and cluster 2. (B) Heatmap of the top five genes highly expressed in NK cell clusters. (C) The specific markers expression of NK cells in UMAP plot. (D) Pseudotime-ordered analysis of KIR3DL2^high^ NK cells from HCC tumor tissue. (E) Diagram illustrating the expression of specific markers at different stages of NK cell differentiation.

According to our single-cell dimensionality reduction clustering, KIR3DL2 expression was predominantly observed within cluster 0 and cluster 2 NK cell populations (Figures 3A and 3B). To delve into the developmental stage of KIR3DL2-expressing NK cells, we analyzed specific markers within distinct NK cell subclusters. Notably, NK cells exhibiting high KIR3DL2 expression concurrently displayed elevated levels of FCGR3A (CD16) and reduced expression of NCAM1 (CD56) (Figure S2A). Employing classical classification criteria, we categorized NK cells expressing KIR3DL2 as mature NK cells. Our analysis of classical NK cell markers revealed that those with heightened KIR3DL2 expression partially expressed KLRC1, NCR1, IL-RB1, CCR7, KLRG1, and SELL, and prominently expressed KLRD1, while B3GAT1 PDCD1 and CTLA4 was absent. Intriguingly, NK cells exhibiting elevated KIR3DL2 expression did not manifest the conventional mature NK cell activation markers IFNG and tumor necrosis factor (TNF) (Figures 3C and S2B). Furthermore, employing single-cell pseudo-temporal analysis via “monocle” revealed that NK cells exhibiting heightened KIR3DL2 expression were positioned in an early differentiation stage among infiltrating NK cells within tumor tissues (Figure 3D). Based on these comprehensive findings, we tentatively propose that NK cells exhibiting high KIR3DL2 expression in liver tumors predominantly signify differentiated mature NK cells (Figure 3E). Nevertheless, these cells do not demonstrate the characteristic functions associated with activated NK cells. Therefore, we categorize them as “Reserve NK cells” or “NK cells in a quiescent state.”

### Functional insights of KIR3DL2-Expressing NK cells in tumor microenvironment: Cell-cell interactions and signaling pathways

**Figure 4:**
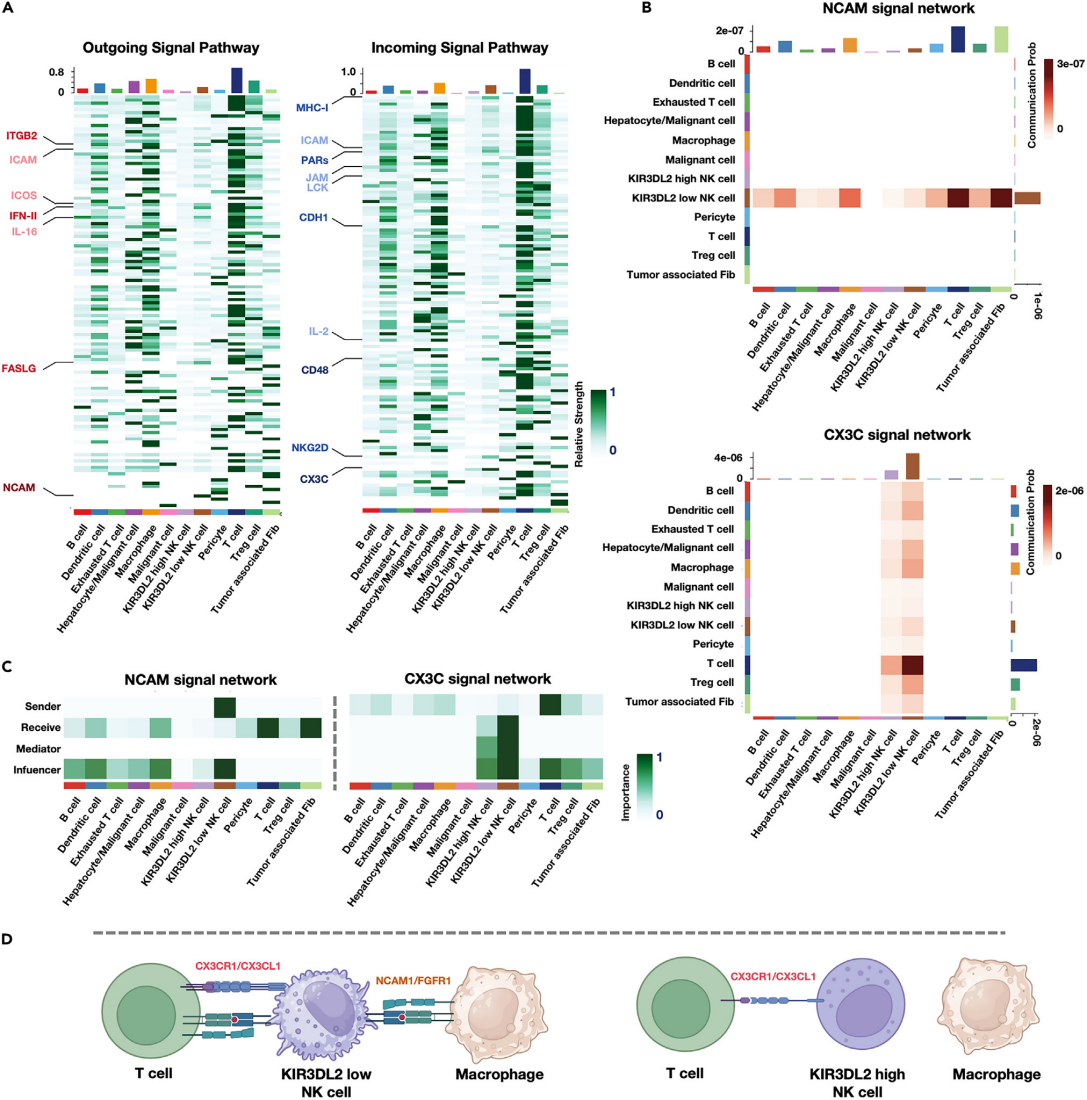
Differences in signaling pathways and cell communication between NK cells with high and low expression of KIR3DL2 (A) Differences in outgoing signal pathways and incoming signal pathways between KIR3DL2^high^ and KIR3DL2^low^ NK cells. (B) The differences in cell communication between KIR3DL2^high^ and KIR3DL2^low^ NK cells in the NCAM1 signaling pathway and CX3C signaling pathway with other cells. (C) The roles played by KIR3DL2^high^ and KIR3DL2^low^ NK cells in the NCAM1 signaling pathway and CX3C signaling pathway. (D) Illustration of the cell communication differences between KIR3DL2^high^ and KIR3DL2^low^ NK cells.

Based on our findings, we sought to delve deeper into the interactions of NK cells expressing high levels of KIR3DL2, suggesting a quiescent state, with other immune or tumor cells within the tissue microenvironment. Utilizing “CellChat,”^18^ a single-cell data analysis tool designed for investigating cellular interactions and communication, we segregated NK cells into subgroups based on high and low KIR3DL2 expression. We investigated alterations in outgoing and incoming signal pathways within both subgroups (Figures 4A and 4B). The results indicated that NK cells displaying elevated KIR3DL2 expression showed minimal activation in both outgoing and incoming signal pathways, displaying scarce interactions with neighboring cells. Conversely, NK cells with diminished KIR3DL2 expression exhibited significant activation, notably within the NCAM, IFN-II, and ITGB2 pathways among the outgoing signal pathways. Particularly in the NCAM pathway, NK cells with reduced KIR3DL2 expression acted as signaling emitters, engaging prominently with tumor-infiltrating macrophages, T cells, and tumor-associated fibroblasts, in which process, those NK cells mainly exert intercellular communication functions through the interaction of NCAM1-FGFR1 receptor-ligand (Figure S2C). Additionally, within the incoming signal pathway, conspicuous differences emerged in the CX3C signaling pathway between NK cell subsets with high and low KIR3DL2 expression. NK cells with reduced KIR3DL2 expression functioned as signal recipients, presenting remarkable activation in the CX3C pathway (Figure 4C). This observation may suggest a pivotal role of the CX3CL1-CX3CR1 axis in regulating the depletion of reservoir NK cells within the tumor microenvironment (Figure 4D). Subsequent analysis using TCGA database highlighted a significant correlation between CX3CL1 expression in tumor tissues and patient prognoses (Figure S2D).

### Low expression of KIR3DL2 was associated with poor prognosis of HCC patients

**Figure 5:**
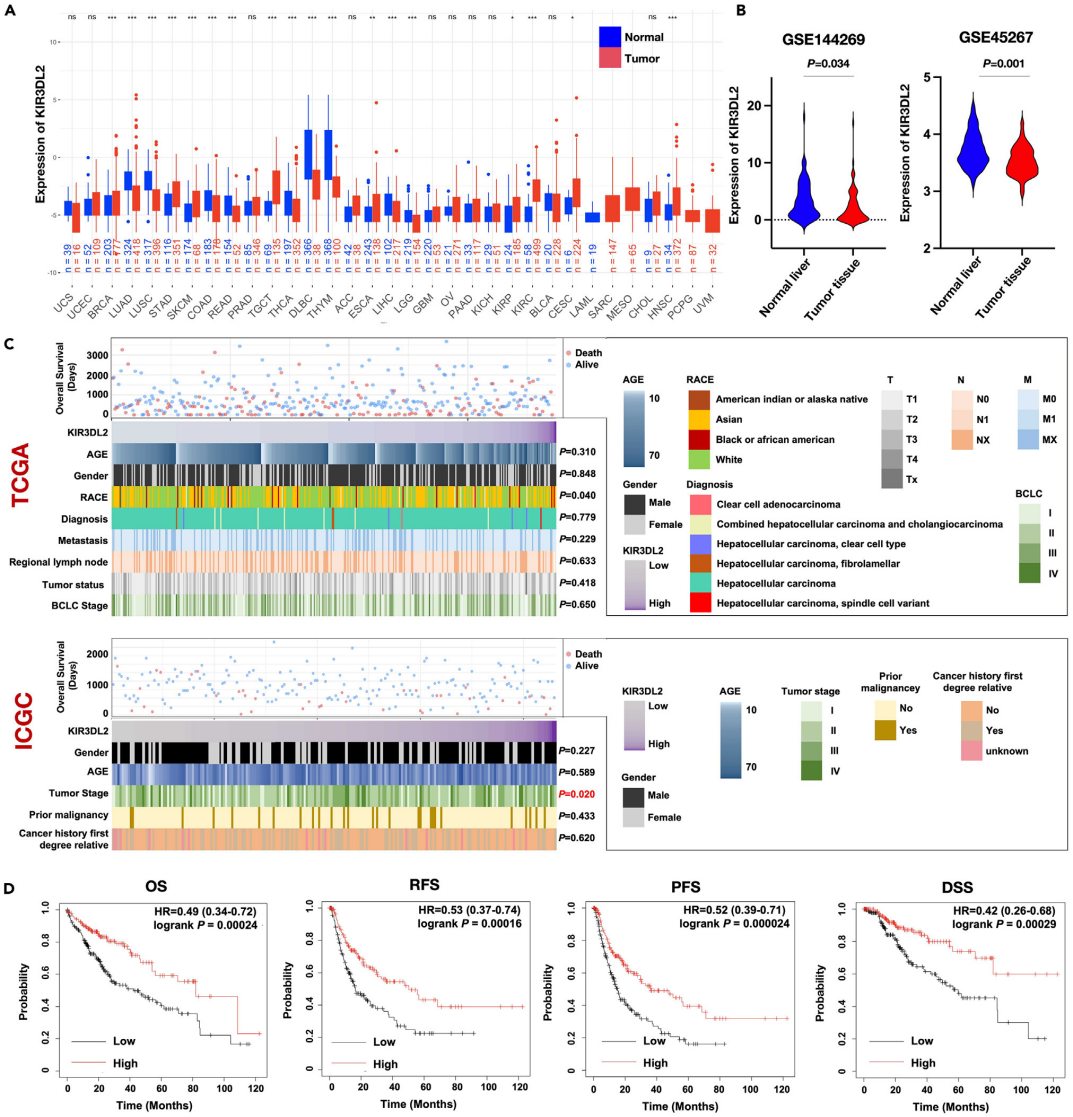
The expression of KIR3DL2 in HCC and its clinical significance in determining prognosis of HCC patients (A) The expression profile of KIR3DL2 in tumor and adjacent non-tumor tissues across various cancers. ∗:*p* < 0.05; ∗∗:*p* < 0.01; ∗∗∗: *p* < 0.001. (B) The different expression of KIR3DL2 in tumor and adjacent non-tumor tissues from Geo database. (C) The landscape of clinical feature of HCC patients related to KIR3DL2 in TCGA and ICGC database. (D) Kaplan-Meier analysis of KIR3DL2 for survival of HCC patients from TCGA database.

Further investigation into the impact of KIR3DL2 on tumor progression led us to conduct a comprehensive study of this molecule using RNA sequencing (RNA-seq) data from multiple datasets of liver tissues. Initially, we analyzed the expression of KIR3DL2 across various tumor tissues and their matched normal tissues obtained from TCGA and the Genotype-Tissue Expression (GTEx) databases. The analysis revealed a significant downregulation of KIR3DL2 in several tumor types, including lung adenocarcinoma, non-small cell lung cancer, diffuse large B-cell lymphoma, and thymoma (Figure 5A). Subsequently, utilizing data from NCBI Geo Databases (GSE144269 and GSE45267) containing matched liver and liver tumor tissues, we corroborated our earlier findings from single-cell sequencing analysis, showing markedly lower expression of KIR3DL2 within liver tumor tissues compared to normal liver tissues (Figure 5B).

Furthermore, we collected clinical data from 372 cases in TCGA database and 215 cases in the International Cancer Genome Consortium (ICGC) database of HCC patients. Correlation analyses between patient clinical characteristics and KIR3DL2 expression revealed few significant associations with general clinical features. However, a notable correlation was found between KIR3DL2 expression and tumor staging in patients from the ICGC database (*p* = 0.02) (Figure 5C). Subsequent Kaplan-Meier plotter analysis^19^ demonstrated a significant correlation between KIR3DL2 expression and various prognostic indicators among HCC patients. Patients exhibiting lower KIR3DL2 expression levels displayed poorer prognosis, as evidenced by reduced overall survival (OS), recurrence-free survival (RFS), progression-free survival (PFS), and disease-specific survival (DSS) rates, indicating a higher tendency for tumor recurrence and mortality (Figure 5D).

### Functional insights and immune regulatory roles of KIR3DL2 in HCC

**Figure 6:**
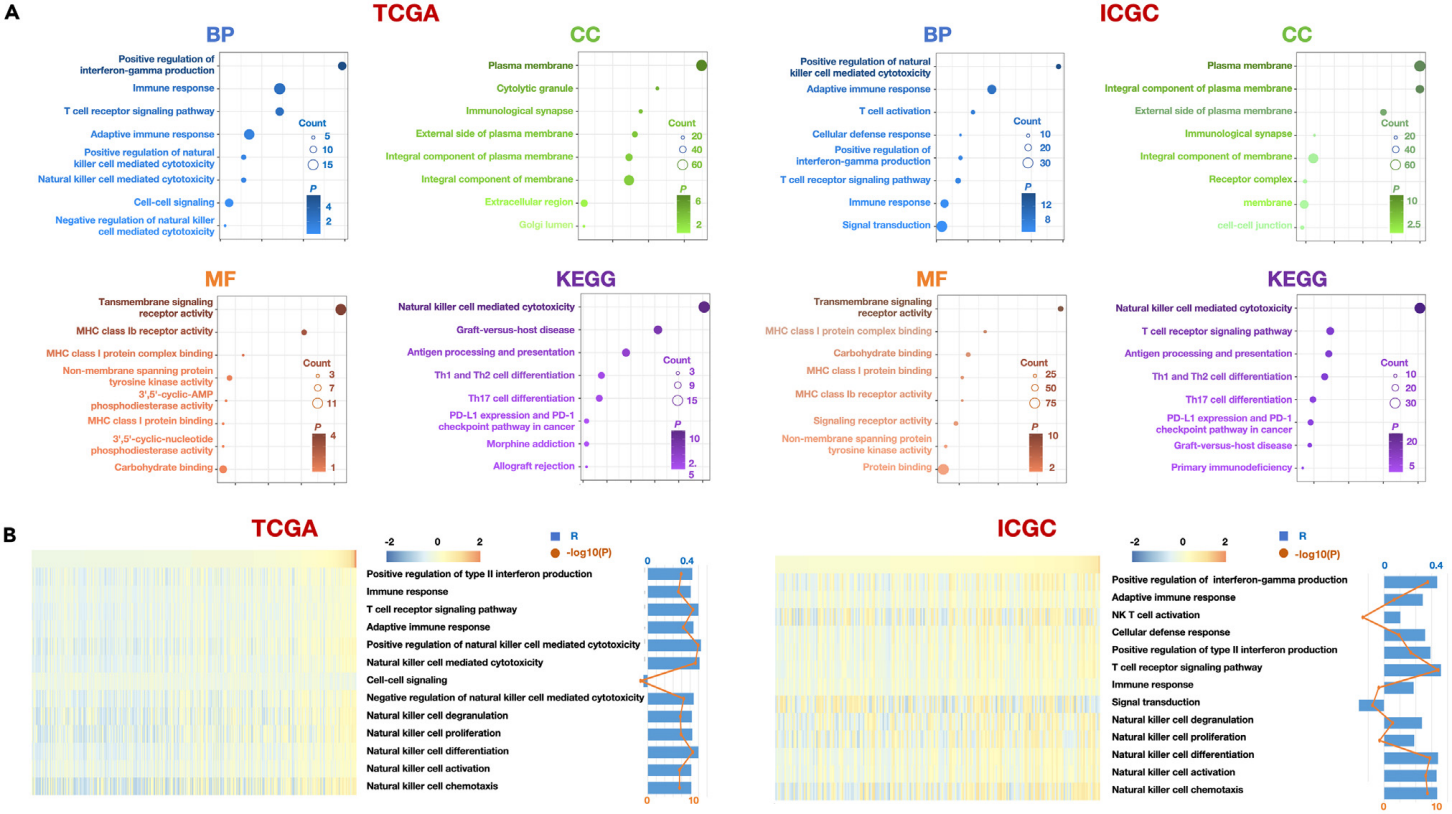
The biological process analysis of KIR3DL2 (A) MF, BP, CC, and KEGG are mostly related to KIR3DL2 in TCGA and ICGC database. (B) Correlation analysis between KIR3DL2 expression and related biological processes enrichment scores.

To delve deeper into the cellular and biological functions of KIR3DL2, we conducted Pearson correlation analysis to identify genes significantly associated with KIR3DL2 in both TCGA and ICGC databases (|R| > 0.5, *p* < 0.05). Subsequently, we subjected these enriched genes to thorough Gene Ontology (GO) and Kyoto Encyclopedia of Genes and Genomes (KEGG) analyses. In TCGA dataset, the biological processes (BP) linked to KIR3DL2 primarily involved the positive regulation of interferon-gamma production, while in the ICGC dataset, they were primarily associated with the positive regulation of NK cell-mediated cytotoxicity. Notably, these processes are closely intertwined with the functionality of NK cells. Additionally, we observed that the plasma membrane emerged as the cellular component (CC) most strongly correlated with KIR3DL2. The molecular functions (MF) attributed to KIR3DL2, as discerned from analyses of both databases, were characterized as transmembrane signaling receptor activity. Moreover, the KEGG pathway analysis from both datasets revealed a compelling correlation between KIR3DL2 and the NK cell-mediated cytotoxicity pathway. These cumulative findings strongly suggest a substantive relationship between KIR3DL2 and the functionality of NK cells within HCC tissues, indicating a pivotal role in regulating NK cell immunity and influencing HCC progression (Figure 6A). Additionally, we conducted GO and KEGG analyses specifically focused on KIR3DL2 in NK cells using single-cell data. The outcomes revealed that within NK cells, KIR3DL2 was predominantly associated with the biological process of cytoplasmic translation. Moreover, the CC most strongly correlated with KIR3DL2 was the external side of the plasma membrane, and its primary molecular function was identified as transmembrane signaling receptor activity. The KEGG analysis highlighted that the most predominant signaling pathways associated with KIR3LD2 were antigen processing and presentation, along with NK cell-mediated cytotoxicity (Figure S3).

Based on the aforementioned findings, we employed gene set variation analysis (GSVA) to assess the enrichment scores of KIR3DL2 in immune regulation. The GSVA results from TCGA and ICGC databases indicated a significant positive correlation between KIR3DL2 and the regulation of NK cell differentiation, activation, chemotaxis, and IFN-gamma secretion (Figure 6B). Besides, it displayed some association with T cell receptor signaling pathways (Figure 6B). This finding corresponds with our earlier observation of the CX3CL1-CX3CR1 receptor-ligand signaling pathway activation obtained through CellChat analysis.

Additionally, we conducted a correlation analysis of specific genes closely linked to the functional aspects of NK cells and KIR3DL2. This analysis encompassed NK cell adhesion molecules, inhibitory receptors, activating receptors, cytokine and chemokine receptors, as well as death receptors. The results highlighted a significant correlation between KIR3DL2 and inhibitory receptors, along with a certain degree of correlation with FASLG expression (Table S3). Moreover, we performed correlation analyses on gene sets related to several essential cytokines secreted by NK cells and genes sets associated with MHC-I molecule binding function. The findings suggested a notable correlation between KIR3DL2 and MHC-I molecule binding as well as IFN secretion by NK cells (Figure S4).

### Investigation of KIR3DL2 expression on NK cells in HCC patients

**Figure 7:**
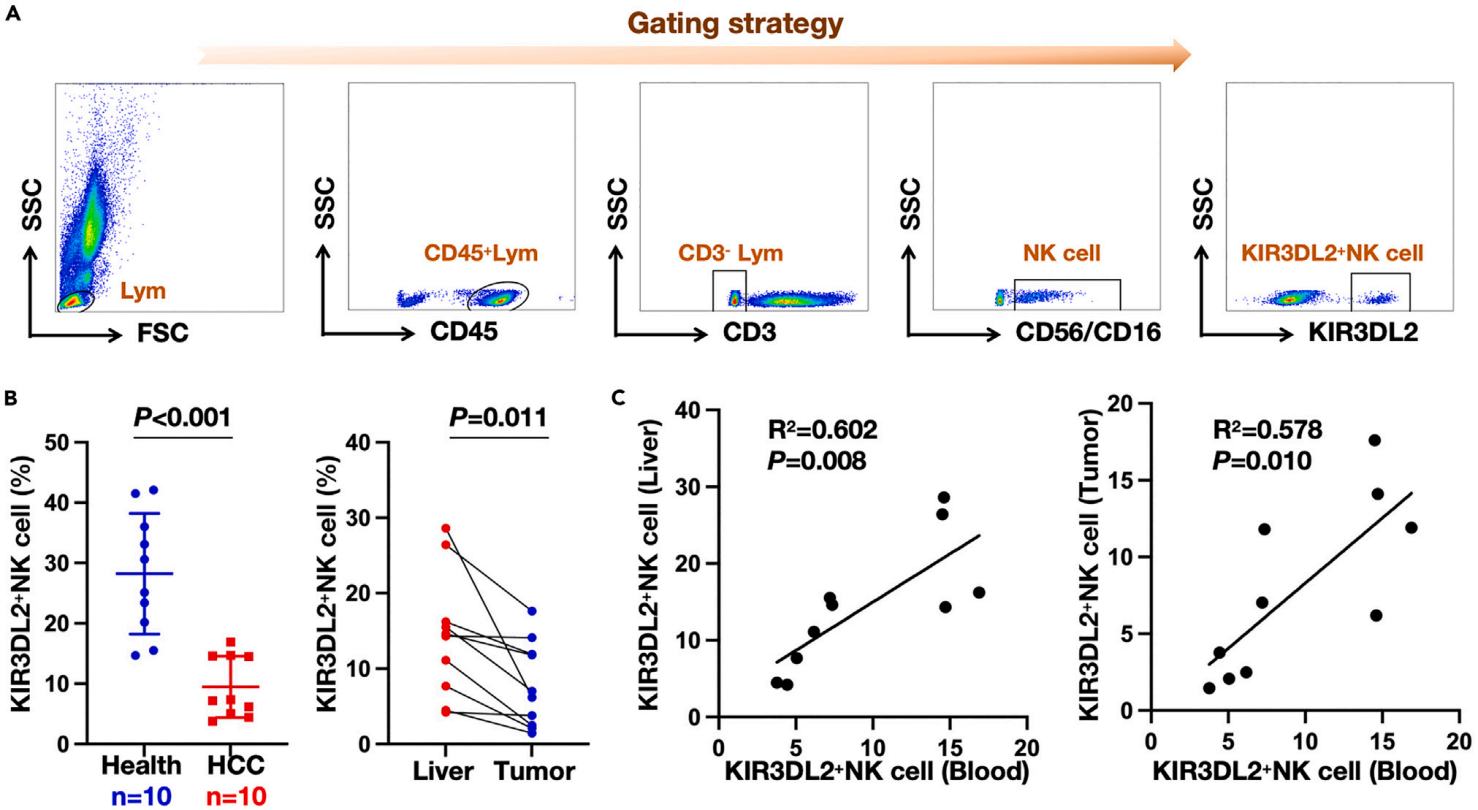
Flow cytometry detection of KIR3DL2 expression on NK cells in peripheral blood, adjacent tissues, and tumor tissues of HCC patients (A) Flow cytometry gating strategy. (B) Expression of KIR3DL2 on NK cells in peripheral blood of healthy individuals and HCC patients, as well as in adjacent and tumor tissues from HCC patients. (C) The correlation between KIR3DL2 expression on NK cells in peripheral blood and in adjacent and tumor tissues.

To further authenticate KIR3DL2 expression on NK cells within HCC patients, we assembled a small cohort involving peripheral blood samples from 10 HCC patients along with matched samples from healthy controls. Complementary to this, we procured paired samples of tumor tissues and corresponding adjacent non-tumor tissues from the HCC patients. Using flow cytometry, we evaluated KIR3DL2 expression on infiltrating NK cells in both peripheral blood and liver tissues (Figure 7A). The results exhibited notably higher KIR3DL2 expression on NK cells in the peripheral blood of healthy individuals than those diagnosed with HCC. Conversely, KIR3DL2 expression on infiltrating NK cells within the tumor tissues of HCC patients was lower compared to their paired adjacent non-tumor tissues (Figure 7B). Additionally, a correlation analysis was performed to assess the relationship between KIR3DL2 expression on NK cells in peripheral blood and liver tissues. The findings revealed a significant correlation between KIR3DL2 expression on NK cells in peripheral blood and its expression in both adjacent non-tumor and tumor tissues (Figure 7C). These outcomes suggest a potential association, indicating that peripheral blood NK cells might serve as a surrogate for evaluating KIR3DL2 expression on NK cells within tumor tissues, offering promise in prognosticating patient outcomes. Additional, pervious research has reported its limited expression on T cells,^20^^,^^21^ and we have also verified this in experiments. The results showed that there is a small amount of KIR3DL2 expression on CD4^+^ and CD8^+^ cells (Figure S5).

## Discussion

The liver harbors abundant immune cells. NKcells, as crucial components of innate immunity, play a significant role in the body’s anti-tumor processes. Several studies have indicated that NK cells can effectively kill tumor cells in the early stages of liver tumor development. However, as the disease progresses, the number of infiltrating NK cells in the tumor gradually decreases, and the loss of NK cells becomes a crucial factor in immune dysregulation and tumor progression.^22^^,^^23^ In this study, we also observed through single-cell data that in the majority of HCC patients, the infiltrating NK cells within the tumor tissue were significantly lower compared to adjacent non-tumor tissue. Furthermore, patients who experienced recurrence exhibited even lower levels of NK cells in their tumors compared to those who did not experience recurrence.

Activated NK cells can eliminate tumor cells through various pathways. For instance, NK cells can rapidly establish connections with tumor cells through integrins and then induce apoptosis in target cells by disrupting the cell membrane structure with granule enzymes and perforin. Alternatively, they can utilize the death receptor pathways of Fas ligand and TNF-related apoptosis-inducing ligand (TRAIL) to kill tumor cells.^24^ However, the prerequisite for all these mechanisms is that the inhibitory receptors on the surface of mature NK cells are not engaged with the MHC-I molecules of target cells.^25^ In the study, we identified an inhibitory receptor belonged to the KIR family, KIR3DL2, which is significantly downregulated in NK cells infiltrating tumor tissues of HCC patients. Moreover, patients who experienced recurrence exhibited significantly lower expression levels of KIR3DL2 in NK cells compared to those who did not experience recurrence. Several studies have already reported that KIR3DL2 can recognize the allogeneic forms of HLA-B27, HLA-A3, and HLA-A11. Upon binding to the target epitope, phosphorylation occurs in the intracellular immunoreceptor tyrosine-based inhibition motif (ITIM) of KIR3DL2, recruiting the inhibitory phosphatases SHP1 and SHP2 to the signaling complex. This mechanism inhibits NK cell attacks on normal cells.^26^^,^^27^ So, theoretically, KIR3DL2^high^ NK cells should exhibit strong cytolytic and inflammatory responses against cells with reduced surface expression of self-HLA-A. However, our experimental results revealed that, although KIR3DL2^high^ NK cells belong to the mature NK cell subset characterized by the CD56^low^CD16^high^ phenotype, they do not internally express IFN-γ and TNF-α. Additionally, NK cells with high KIR3DL2 expression exhibit elevated levels of inhibitory receptors KLRC1 and KLRD1 (NKG2A/CD94), and low or no expression of terminal differentiation markers B3GAT1 (CD57) and KLRG1, alone with exhaustion markers PDCD1 and CTLA4. As tissue-infiltrating NK cells, those with high KIR3DL2 expression also specifically express tissue-resident NK (trNK) cell markers CCR7 and SELL (CD62L).^28^^,^^29^^,^^30^^,^^31^^,^^32^ Besides, pseudotemporal analysis and CellChat results suggest that cells with high expression of KIR3DL2 are in the early stages of differentiation among liver-infiltrating NK cells and exhibit no significant interactions with other cells. We refer to this subset of NK cells with high KIR3DL2 expression in liver tissues as quiescent NK cells or reserve NK cells. Therefore, we speculate that the reduction of KIR3DL2 expression on NK cell in the tumor tissue of HCC patients who experience recurrence indicates insufficient reserves of NK cells within the tumor, with the majority of NK cells in an exhausted state and a significantly diminished ability to eliminate the tumor cells. Consequently, this increases the likelihood of tumor recurrence and progression.

Finally, our study on the expression of KIR3DL2 in peripheral blood and tissues reveals a strong correlation between KIR3DL2 expression on peripheral blood NK cells and tissue-infiltrating NK cells. Combining this with Kaplan-Meier results from TCGA database regarding KIR3DL2 in HCC patients, we propose that assessing KIR3DL2 expression on peripheral blood NK cells could serve as a potential prognostic marker for HCC patients. However, to confirm this, further in-depth research involving a large cohort of individuals is warranted.

### Limitations of the study

This study has several limitations. First, we did not perform further functional assays to validate the impact of KIR3DL2 on NK cells, nor did we elucidate the pathways through which KIR3DL2 affects NK cell function and its subsequent impact on tumors. Second, the number of HCC patients included in our preliminary study is insufficient to validate whether KIR3DL2 can effectively predict the prognosis of HCC patients in a large cohort. Future studies will refine the experimental design and further explore the specific molecular mechanisms of KIR3DL2 in HCC.

## Resource availability

### Lead contact

Further information and requests about this study should be directed to and will be fulfilled by the lead contact, Beili Wang (wang.beili1@zs-hospital.sh.cn).

### Materials availability

This study did not generate new unique reagents.

### Data and code availability

• All single-cell sequencing data and RNA-seq data can be downloaded from GEO and the China National GeneBank Database. Accession numbers are listed in the key resources table.
• This paper does not report original code.
• Any additional information required to reanalyze the data reported in this paper is available from the lead contact upon request.

## Acknowledgments

We would like to thank the providers of public database bioinformation used in this study.

This research was funded by the 10.13039/501100001809National Natural Science Foundation of China (82202608, 82172348, 81902139, 81972000, and 82102483), the Constructing Project of Clinical Key Disciplines in Shanghai (shslczdzk03302), the Specialized Fund for the Clinical Researches of Zhongshan Hospital, 10.13039/501100003347Fudan University (2020ZSLC54), the Key Medical and Health Projects of Xiamen (YDZX20193502000002), and Shanghai Baoshan Medical Key Specialty (BSZK2023A18).

## Author contributions

B.W., W.Y., and W.G. are responsible for project design; J.Z. and A.J. are responsible for data collection and processing; J.Z. and A.J. are responsible for experiment data verification and analysis; J.Z. and A.J. are responsible for paper writing; B.W., W.Y., and B.P. are responsible for the modification and improvement of the paper. All authors read and approved the final manuscript.

## Declaration of interests

The authors declare no competing interests.

## STAR★Methods

### Key resources table

**Table:** 

REAGENT or RESOURCE	SOURCE	IDENTIFIER
**Antibodies**		

Multitest™ 6-color TBNK	BD Bioscience	Catalog No: 644611
Human KIR3DL2/CD158k Antibody	R&D Systems	Catalog No: MAB2878
Goat anti-Mouse IgG (H + L) Cross-Adsorbed Secondary Antibody, Alexa Fluor™ 488	ThermoFisher Scientific	Catalog No: A-11001
BD Horizon™ BV421 Mouse IgG1, k Isotype Control	BD Bioscience	Catalog No: 562438

**Biological samples**		

HCC patients tumor tissue	Zhongshan hospital, Fudan university	https://www.zs-hospital.sh.cn
HCC patients para-tumor tissue	Zhongshan hospital, Fudan university	https://www.zs-hospital.sh.cn
HCC patients’ blood	Zhongshan hospital, Fudan university	https://www.zs-hospital.sh.cn
Health individuals’ blood	Zhongshan hospital, Fudan university	https://www.zs-hospital.sh.cn

**Deposited data**		

Single-cell sequencing data	China National GeneBank Database	accession number: CNP0000650
RNA-seq sequencing data (371 HCC patients)	TCGA	https://portal.gdc.cancer.gov
RNA-seq sequencing data (251 HCC patients)	ICGC	https://dcc.icgc.org/
RNA-seq results for HCC tissues and matched adjacent non-tumor tissues	NCBI Geo DataSets	GSE144269 and GSE45267

**Software and algorithms**		

R	https://cran.rstudio.com	NA
R studio	https://posit.co/download/rstudio-desktop/	NA
Seurat (v4.3.0)	RRID: SCR_016341; https://github.com/satijalab/seurat	NA
GSVA(v1.47.0)	http://www.bioconductor.org/packages/release/bioc/html/GSVA.html	NA
dplyr (v1.1.0)	https://github.com/tidyverse/dplyr	NA
CellChat (v1.6.1)	https://github.com/sqjin/CellChat	NA
Monocle (2.26.0)	https://www.bioconductor.org/packages/release/bioc/src/contrib/monocle_2.26.0.tar.gz%20tarS-xf%20monocle∗gz/	NA

### Experimental model and study participant details

#### Hepatocellular carcinoma patient and health individuals enrollment

A total of 10 HCC patients who underwent surgical resection and 10 healthy individuals matched for age and gender were recruited between June 2023 and October 2023. All HCC patients were diagnosed with HCC in accordance with the Guidelines for the Diagnosis and Treatment of HCC (2019 Edition). This study has been approved by the Ethics Committee of Zhongshan Hospital, Fudan University, and informed consent has been obtained from all participants involved in the research. The clinical information of the patients and health individuals ars presented in the Tables S1 and S2.

#### Data acquisition

Single-cell sequencing data were obtained from the China National GeneBank Database (CNGVdb) with the accession number CNP0000650. Clinical information, follow-up results, and tissue RNA-seq sequencing data for 371 HCC patients were sourced from the TCGA database (https://portal.gdc.cancer.gov). Additionally, clinical information, follow-up results, and tissue RNA-seq sequencing data for 215 HCC patients were obtained from the ICGC database (https://dcc.icgc.org/). RNA-seq results for HCC tissues and matched adjacent non-tumor tissues (GSE144269 and GSE45267) were retrieved from the NCBI Geo DataSets (https://www.ncbi.nlm.nih.gov/gds/).

### Method details

#### Single-cell sequencing analysis

For the analysis of single-cell sequencing data, we employed the R package (Seurat 4.3.0, dplyr 1.1.0, patchwork 1.1.2, tidyverse 2.0.0). This encompassed various procedures such as data normalization, dimensionality reduction, PCA, Uniform Manifold Approximation and Projection(UMAP)/T-distributed stochastic neighbor embedding (TSNE) clustering, and the identification of differentially expressed genes. R package CellChat (1.6.1), a powerful tool designed for the analysis of cell-cell communication in single-cell data, was utilized to conducted a comprehensive investigation into the intricate networks of cellular interactions within complex biological system, including Cell-Cell communication analysis, ligand-receptor pair analysis, signaling pathway activation and single-cell trajectory analysis. R package Monocle (2.26.0), a widely utilized tool for scRNA-seq analysis, was specifically designed for investigating cellular trajectories and dynamic processes in single-cell datasets. The analysis included data normalization, dimensionality reduction, PCA, UMAP/Tsne clustering, and differential expression analysis.

#### Functional enrichment analysis

The Database for Annotation, Visualization, and Integrated Discovery (DAVID, v2023 q4) was utilized to upload a compilation of the most pertinent genes or cell clusters associated with KIR3DL2. Subsequently, enrichment results from GO analysis KEGG pathway analysis were retrieved. This study presents the top eight genes, arranged in ascending order of *p*-value (*p* < 0.05), based on the obtained results.

#### Gene set variation analysis

GSVA was conducted to assess the functional enrichment of relevant BP and signaling pathways identified through GO analysis. The gene list associated with these processes was obtained from the AmiGO 2 portal (http://amigo.geneontology.org/amigo). Utilizing the R packages (GSVA 1.47.0), the enrichment scores for each HCC sample from the TCGA and ICGC database were computed. The resulting enrichment scores were visualized in a heatmap using the R package (pheatmap 1.0.12). Pearson correlation analysis was performed to investigate the correlation between KIR3DL2 and the identified BP and signaling pathways.

#### Peripheral blood mononuclear cell (PBMC) isolation and flow cytometry testing

Peripheral blood sample processing: Collect 3 mL of peripheral blood from the patient and dilute the sample with 3 mL of PBS (All blood specimens were collected from patients diagnosed with HCC prior to the initiation of any treatment). Slowly layer the diluted peripheral blood sample onto a Ficoll-Paque density gradient centrifuge tube. Through centrifugation, cells in the blood will sediment into the gradient, forming different layers. Collect the PBMCs floating on the upper layer of the Ficoll-Paque for subsequent testing. Tissue sample processing: Rinse the collected liver tissue with PBS and remove surface blood and excess tissue. Use surgical scissors to mince the liver tissue and digest it with Collagenase IV. Filter the digested liver tissue through a 40 μm sieve. Wash the filtered cells with PBS, at least three times. Adjust the cell concentration for further testing. Flow cytometric staining was performed on the extracted PBMCs using CD3-FITC, CD56/CD16-PE, CD45-Percp-Cy5.5, CD4-PE-Cy7, CD19-APC, CD8-APC-Cy7 (644611, BD Bioscience), KIR3DL2 (MAB2878, R&D Systems), and fluorescently conjugated anti-mouse IgG (A-11001, ThermoFisher Scientific). The corresponding isotype antibodies were used as negative controls (562438, BD Bioscience). Flow cytometric analysis of the detection results was conducted using Flowjo 10.6.2 (BD Bioscience).

### Quantification and statistical analysis

SPSS 19.0(IBM) and GraphPad Prism 9.5.0(GraphPad) were applied in this research, Differences between groups were analyzed using two-tailed unpaired Student’s t-test, Pearson’s χ^2^ test, Mann-Whitney U test, two-way ANOVA, or log rank test, as appropriate. A two-tailed *p* < 0.05 were considered statistically significant.

## Supplemental information

Document S1. Figures S1–S5

Table S1. Clinical information of enrolled HCC patients, related to Figure 7

Table S2. Clinical information of enrolled health participants, related to Figure 7

Table S3. The correlation between KIR3DL2 expression and NK cell immunity and inflammatory activities, related to Figure 6
