# Supplementary material for: Exploring the role of KIR3DL2 on NK cells in hepatocellular carcinoma and its potential prognostic implications
Source: iScience. 2024 Aug 8;27(9):110637. doi: 10.1016/j.isci.2024.110637 (PMC11388180; doi:10.1016/j.isci.2024.110637)
Supplement: Document S1. Figures S1–S5 [file mmc1.pdf]

iScience, Volume *27*

Supplemental information

Exploring the role of KIR3DL2 on NK cells in hepatocellular carcinoma and its potential prognostic implications

Jie Zhu, Anli Jin, Baishen Pan, Wei Guo, Wenjing Yang, and Beili Wang

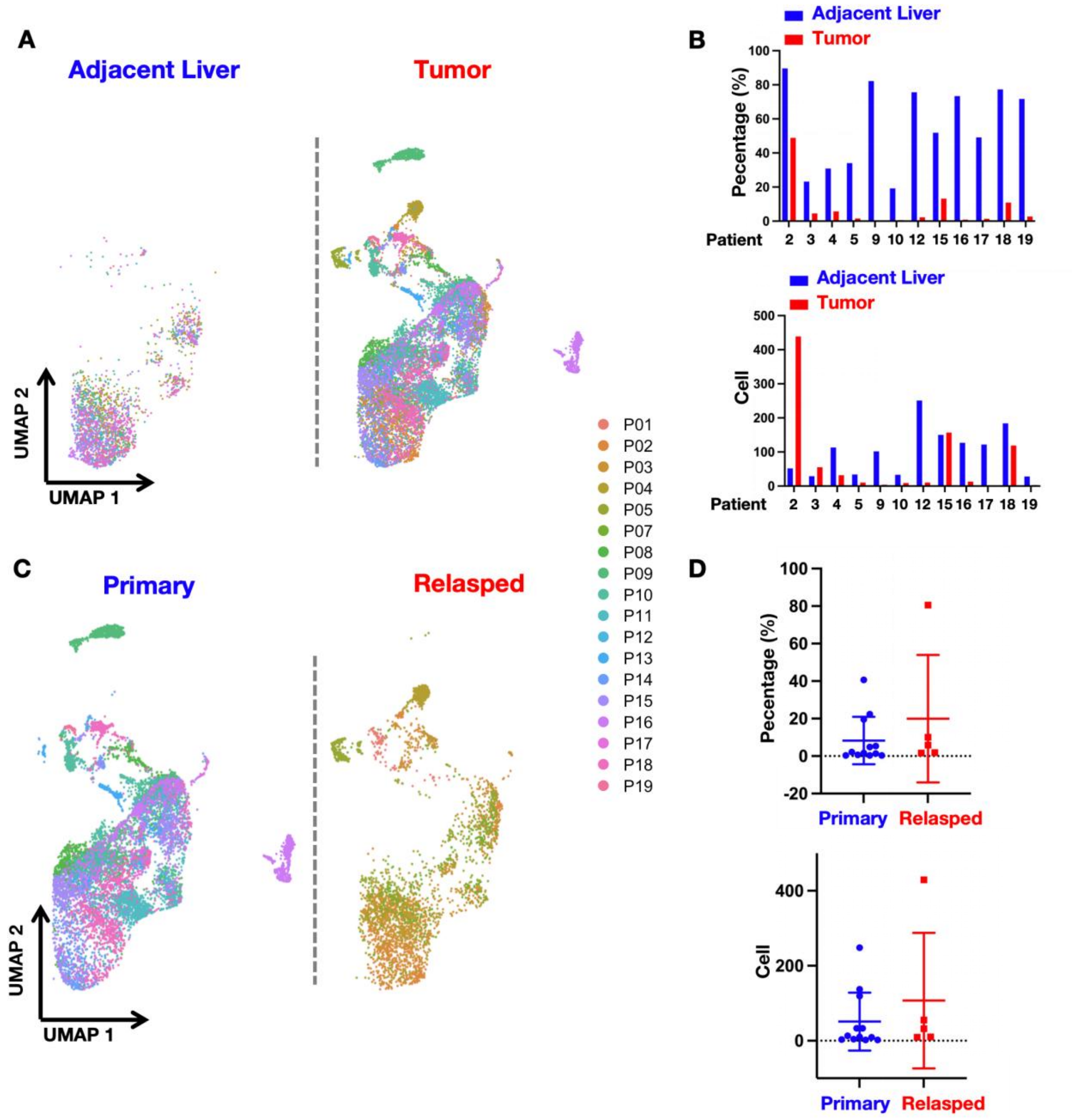

Figure S1. The profile of 19 hepatocellular carcinoma patients single-cell sequencing data. A) Single-cell sequencing data is presented in the form of UMAP plots to show the distribution of HCC patients, split by tumor and adjacent liver. **B)** The number and proportion of cells for each HCC patient in the single-cell sequencing data. **C)** Single-cell sequencing data is presented in the form of UMAP plots to show the distribution of HCC patients, split by recurrence status. **D)** The number and proportion of cells in the primary and recurrent groups of HCC patients. Related to Figure 1.

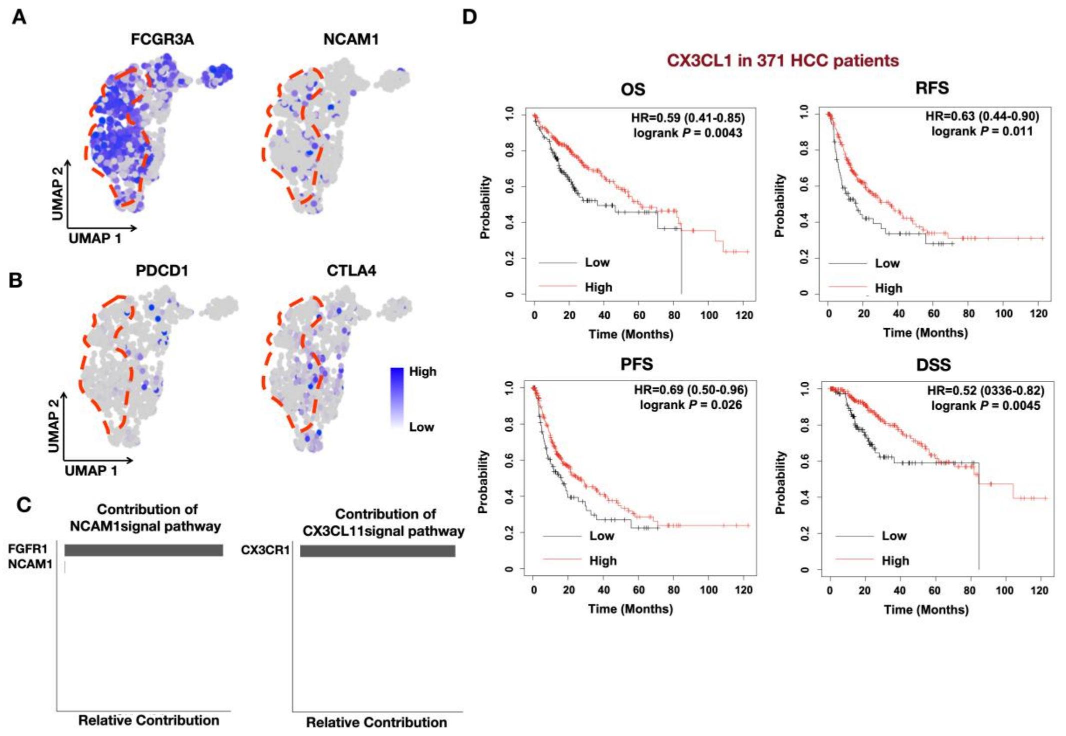

Figure S2. The pattern and cell communication analysis of KIR3DL2^{high} NK cells. **A)** “FCGR3A” and “NCAM1” expression in NK cells. **B)** Exhaustion markers, “PDCD1”and “CTLA4” expression in NK cells. **C)** The relative contribution of NCAM1 and CX3CL1 signal pathway. **D)** Kaplan–Meier analysis of CX3CL1 for survival of HCC patients from TCGA database. Related to Figure 3.

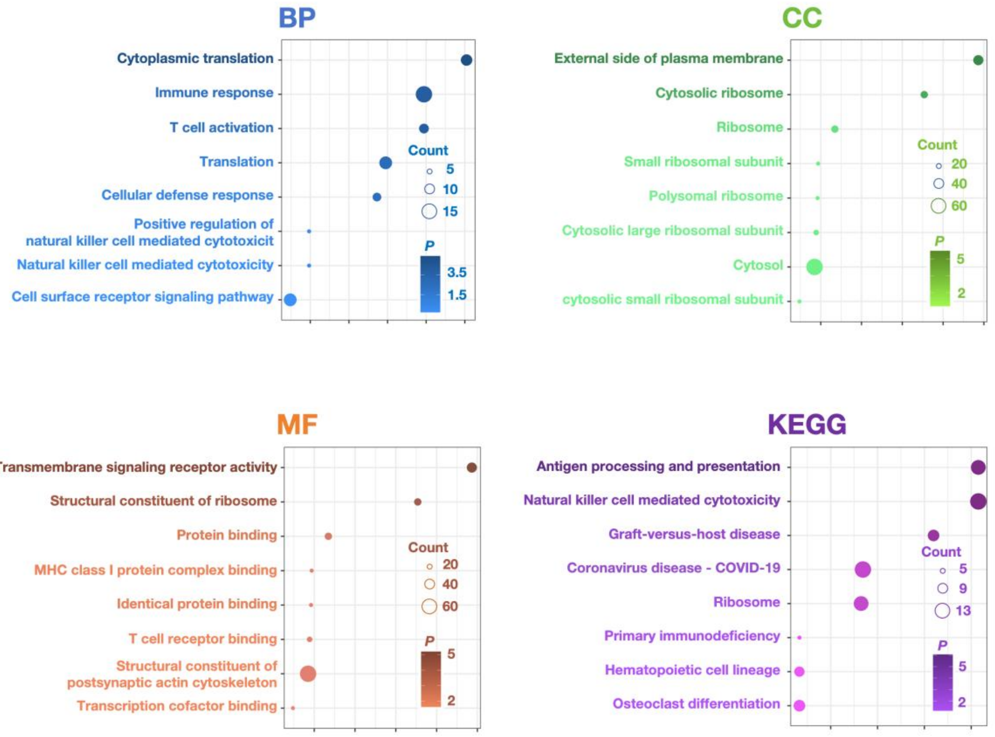

Figure S3. The biological process analysis of KIR3DL2 in NK cluster. Related to Figure 6.

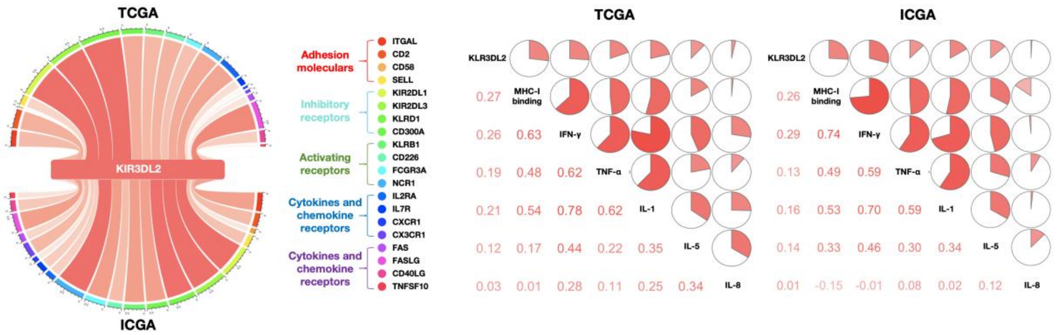

Figure S4. The correlation between KIR3DL2 expression and NK cell biological activities in the TCGA and ICGC databases. Related to Figure 6.

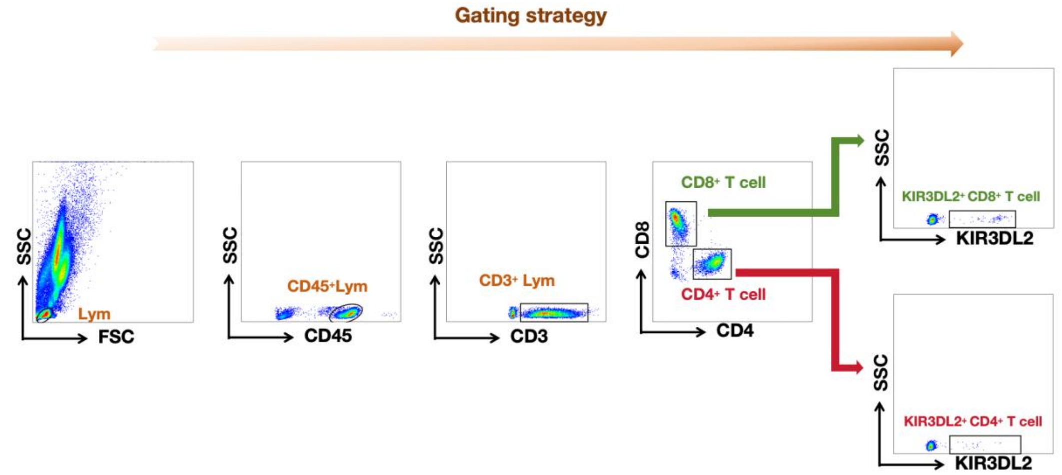

Figure S5. The flow cytometry gating strategy to detect the KIR3DL2 on CD4$^+$ and CD8$^+$ T cells. Related to Figure 7.
